# Streptozotocin, Type I Diabetes Severity and Bone

**DOI:** 10.1007/s12575-009-9000-5

**Published:** 2009-03-06

**Authors:** Katherine Motyl, Laura R McCabe

**Affiliations:** 1 Departments of Physiology and Radiology, Biomedical Imaging Research Center, Michigan State University, 2201 Biomedical Physical Science Bldg., East Lansing, MI 48824, USA

**Keywords:** streptozotocin, Diabetes mellitus, Type I, bone, mice, osteocalcin, adipocytes, osteoporosis

## Abstract

As many as 50% of adults with type I (T1) diabetes exhibit bone loss and are at increased risk for fractures. Therapeutic development to prevent bone loss and/or restore lost bone in T1 diabetic patients requires knowledge of the molecular mechanisms accounting for the bone pathology. Because cell culture models alone cannot fully address the systemic/metabolic complexity of T1 diabetes, animal models are critical. A variety of models exist including spontaneous and pharmacologically induced T1 diabetic rodents. In this paper, we discuss the streptozotocin (STZ)-induced T1 diabetic mouse model and examine dose-dependent effects on disease severity and bone. Five daily injections of either 40 or 60 mg/kg STZ induce bone pathologies similar to spontaneously diabetic mouse and rat models and to human T1 diabetic bone pathology. Specifically, bone volume, mineral apposition rate, and osteocalcin serum and tibia messenger RNA levels are decreased. In contrast, bone marrow adiposity and aP2 expression are increased with either dose. However, high-dose STZ caused a more rapid elevation of blood glucose levels and a greater magnitude of change in body mass, fat pad mass, and bone gene expression (osteocalcin, aP2). An increase in cathepsin K and in the ratio of RANKL/OPG was noted in high-dose STZ mice, suggesting the possibility that severe diabetes could increase osteoclast activity, something not seen with lower doses. This may contribute to some of the disparity between existing studies regarding the role of osteoclasts in diabetic bone pathology. Examination of kidney and liver toxicity indicate that the high STZ dose causes some liver inflammation. In summary, the multiple low-dose STZ mouse model exhibits a similar bone phenotype to spontaneous models, has low toxicity, and serves as a useful tool for examining mechanisms of T1 diabetic bone loss.

## 1. Introduction

Type I (T1) diabetes is associated with many complications including bone loss [1-12]. Approaches to understand the mechanisms accounting for T1 diabetic bone pathology include cell culture models, human clinical studies, and animal models. Cell culture models allow examination of effects of individual factors, such as hyperglycemia, on cell parameters such as signaling, responsiveness, and differentiation. However, tissue pathology is often caused by a combination of factors that are produced by more than one cell type. Clinical studies allow examination of systemic and tissue effects, but are limiting in the types of analyses that can be done. Animal models are a compromise between clinical and in vitro studies. They can be genetically manipulated, their diet/environment can be controlled, and a multitude of analyses can be performed on them, making animal models powerful tools to understand mechanisms of tissue pathology, provided that tissue responses are similar to what is seen clinically.

A variety of T1 diabetic rodent models exist. Some mouse models become spontaneously diabetic, such as the non-obese diabetic (NOD) mice, while other T1 diabetic models are pharmacologically induced by compounds such as alloxan, streptozotocin (STZ), Vacor, Dithizone, and 8-hydroxyquinone (reviewed in [13]). The streptozotocin-induced diabetes model has been extensively used, making it particularly useful for building upon and comparing results of other studies. STZ is a nitrosurea compound derived from *Streptomyces achromogenes*, which has also been used as an antibiotic and a cancer treatment. It enters pancreatic β cells through glucose transporter 2 (GLUT2) channels in the plasma membrane and causes cellular toxicity and local immune responses that lead to hypoinsulinemia and hyperglycemia in animals [14]. In some models, especially rats, a single dose of STZ is effective at inducing T1 diabetes. In mice, however, multiple low doses (40 mg/kg) are the most effective at maintaining mouse viability and inducing pancreatic dysfunction in part through immune destruction. This response is similar to what is seen during the onset of T1 diabetes in humans [14], although lymphocytes are not required for STZ induction of diabetes [15]. The benefits of the STZ-induced diabetic mouse model include that it allows one to induce diabetes in genetically altered mice, maintain mice in a controlled environment, regularly monitor and directly measure serum and bone factors, obtain bone samples for high-resolution analyses, and chose the time of diabetic induction (compared to waiting for the diabetes to occur in spontaneous models).

Examination of bones from STZ-induced diabetic mice demonstrates a significant decrease in bone volume fraction marked by decreased levels of markers of osteoblast maturation and increased levels of markers of adipogenesis [16]. However, to confirm that this model is appropriate to study T1 diabetic bone pathology, it is important to prove that the STZ-injected mice exhibit a bone phenotype similar to spontaneously T1 diabetic mice and, most importantly, similar to T1 diabetic humans. Indeed, low multiple low-dose STZ induction of diabetes causes a bone phenotype consistent with human studies [1-12] and spontaneous mouse models such as NOD mice [17], confirming the utility of the STZ model for studying mechanisms of T1-diabetes-induced bone loss.

While STZ-induced diabetes produces consistent bone pathologies (i.e., decreased bone density, increased marrow adiposity), we have found that the magnitude of effects can vary (being greater in some experiments compared to others). We hypothesized that this difference may be related to the activity/strength of the STZ that we inject. Manufacturer (Sigma) data sheets indicate that STZ is stable for approximately 2 years if frozen (-20°C) and protected from the light. In solution, STZ is stable around a pH of 4 and therefore is prepared in cold citrate buffer at a pH of 4.5 to enhance stability. If not used fresh, STZ solutions can exhibit reduced ability to induce diabetes.

In this study, we examined the role of STZ concentration (40 or 60 mg/kg) in multiple dose induction of diabetes, disease severity, and bone loss. Both doses caused a similar level of bone loss, but the higher dose of STZ caused a more rapid elevation of blood glucose levels, greater magnitude of change in body mass, fat pad mass, and bone gene expression (osteocalcin, aP2). While markers of kidney function appeared normal in all conditions, liver inflammation was detected in the high-STZ-dosed mice. An increase in bone cathepsin K and in the RANKL/osteoprotegrin (OPG) ratio was noted only in the high-dose group, suggesting that increased diabetes severity may lead to increased osteoclast activity. This may explain some of the disparity between existing studies regarding the role of osteoclasts in diabetic bone pathology.

## 2. Materials and Methods

### 2.1. STZ Induction of Diabetes

Male BALB/c mice (Harlan Laboratories, Houston, TX, USA), 10 weeks old, were given daily intraperitoneal injections with STZ (40 or 60 μg/g body weight in 0.1 M citrate buffer) for 5 days to induce diabetes [14,18]. Control mice were injected with buffer alone. Blood glucose levels were examined 7 days after the final streptozotocin injection by obtaining blood from the lateral saphenous vein and measuring glucose concentration with a glucometer (Accu-Check instant, Boehringer Mannheim Corporation, Indianapolis, IN, USA). Mice with blood glucose levels greater than 300 mg/dl were considered diabetic. At the time of harvest, 19 days after the first streptozotocin injection, blood glucose measurements were again obtained along with total body, tibialis anterior, and subcutaneous femoral fat pad mass. Animal studies were conducted in accordance with the Michigan State University Institutional Animal Care and Use Committee.

### 2.2. Serum Assays

Blood was collected at the time of harvest, allowed to clot at room temperature for 5 min, then centrifuged at 4,000 rpm for 10 min. Serum was removed and stored at -80°C. Serum went through no more than two freeze/thaw cycles. Serum osteocalcin was measured using a mouse osteocalcin EIA kit (BT-470, Biomedical Technologies, Inc., Stoughton, MA, USA) according to the manufacturer's protocol. Serum TRAP5b was measured using a MouseTRAP assay kit (SB-TR103, Immunodiagnostic Systems Inc., Fountain Hills, AZ, USA) according to the manufacturer's protocol.

Serum blood urea nitrogen (BUN), creatinine, and 25-hydroxyvitamin D were measured at the Diagnostic Center for Population and Animal Health (DCPAH) at Michigan State University. Because of volume requirements for assays at DCPAH, serum samples had to be pooled. For BUN and creatinine, two samples were submitted for control and 40-mg/kg dose and one sample was submitted for the 60-mg/kg dose (each submitted sample contained serum from five different mice). For 25-hydroxyvitamin D, three samples were submitted for control, 40 mg/kg, and 60 mg/kg groups (each submitted sample contained serum from one to four mice).

### 2.3. Mineral Apposition Rate

Mice were injected intraperitoneally with 200 μl of 10 mg/ml calcein (Sigma, St. Louis, MO, USA) dissolved in saline 7 and 2 days before harvest. L3 vertebrae were fixed in formalin at time of harvest then transferred to 70% ethanol after 24 h. Vertebrae were then embedded and sectioned at 5 μm on a Reichert Jung 2030 rotary microtome. Sections were photographed under fluorescent light and the distance between lines of calcein was measured.

### 2.4. Micro-computed Tomography Analyses

Fixed bones were scanned with a calibration standard using a GE Explore Locus micro-computed tomography system at a voxel resolution of 20 μm. A threshold of 1,400 was used to separate bone from bone marrow. Trabecular bone analyses were made in a region of trabecular bone defined at 0.17 mm under the growth plate of the tibia extending 2 mm toward the diaphysis and excluding the outer cortical shell. Bone parameters were computed by a GE Healthcare MicroView software application for visualization and analysis of volumetric image data.

### 2.5. RNA Analysis

Whole tibiae (or liver) were crushed under liquid nitrogen conditions using a Bessman tissue pulverizer and homogenized (Omni International TH homogenizer, Marietta, GA, USA) in TRI reagent solution (Molecular Research Center, Inc., Cincinnati, OH, USA) and RNA was extracted. RNA integrity was verified by formaldehyde-agarose gel electrophoresis. Synthesis of complementary DNA (cDNA) was performed by reverse transcription with 2 μg of total RNA using the Superscript II kit with oligo dT(_12–18_) primers according to the manufacturer's protocol (Invitrogen, Carlsbad, CA, USA). cDNA (1 μl) was amplified by polymerase chain reaction (PCR) in a final volume of 25 μl using the iQ SYBR Green Supermix (Bio-Rad, Hercules, CA, USA) with 10 pmol of each primer (Integrated DNA Technologies, Coralville, IA, USA). Osteocalcin was amplified using 5'-ACG GTA TCA CTA TTT AGG ACC TGT G-3' and 5'-ACT TTA TTT TGG AGC TGC TGT GAC-3' [19]. Adipocyte fatty-acid-binding protein 2 (aP2) was amplified using 5'-GCG TGG AAT TCG ATG AAA TCA-3' and 5'-CCC GCC ATC TAG GGT TAT GA-3' [20]. TRAP5 was amplified using 5'-AAT GCC TCG ACC TGG GA-3' and 5'-CGT AGT CCT CCT TGG CTG CT-3' [21]. Cathepsin K was amplified using 5'-GCA GAG GTG TGT ACT ATG-3' and 5'-GCA GGC GTT GTT CTT ATT-3' [22]. RANKL was amplified using 5'-TTT GCA GGA CTC GAC TCT GGA G-3' and 5'-TCC CTC CTT TCA TCA GGT TAT GAG-3' according to Zhao et al. [23]. OPG was amplified using 5'-GAA GAA GAT CAT CCA AGA CAT TGA C-3' and 5'-TCC ATA AAC TGA GTA GCT TCA GGA G-3'. IL-1β was amplified using 5'-CAG GAT GAG GAC ATG AGC ACC-3' and 5'-CTC TGC AGA CTC AAA CTC CAC-3' [24]. IL-6 was amplified using 5'-ATC CAG TTG CCT TCT TGG GAC TGA-3' and 5'-TAA GCC TCC GAC TTG TGA AGT GGT-3'. Hypoxanthine–guanine phosphoribosyl transferase (HPRT), which was not modulated under diabetic conditions, was used as a control for RNA levels; it was amplified using 5'-AAG CCT AAG ATG AGC GCA AG-3' and 5'-TTA CTA GGC AGA TGG CCA CA-3' and exhibited similar kinetics of amplification compared to other genes examined. Real-time PCR was carried out for 40 cycles using the iCycler (Bio-Rad) and data were evaluated using the iCycler software. Each cycle consisted of 95°C for 15 s, 60°C for 30 s (except for osteocalcin which had an annealing temperature of 65°C), and 72°C for 30 s. cDNA-free samples, a negative control, did not produce amplicons. Melting curve and gel analyses (sizing, isolation, and sequencing) were used to verify single products of the appropriate base pair size.

### 2.6. Statistical Analyses

All statistical analyses were performed using Microsoft Excel data analysis program for *t* test analysis. Values are expressed as a mean ± SE.

## 3. Results and Discussion

### 3.1. STZ Injection (40 and 60 mg/kg) Effectively Induces Type I Diabetes in BALB/c Mice

**Figure 1:**
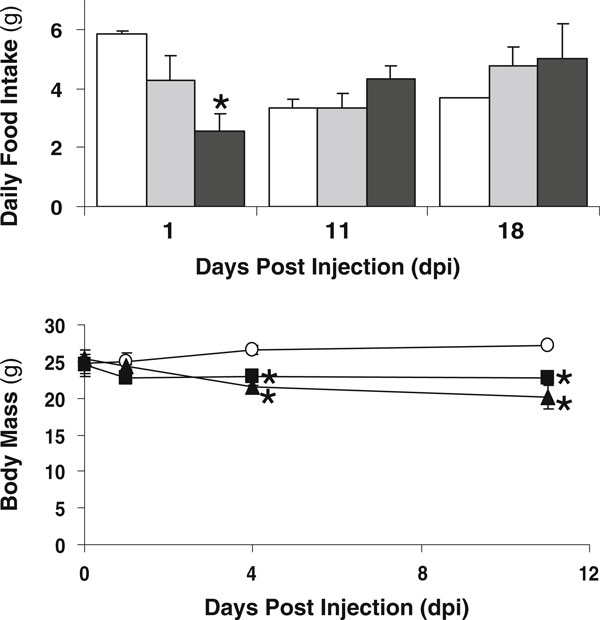
**Mice exhibit decreased food intake following the first STZ injection and lose weight despite becoming hyperphagic at later time points**. *Top panel*, Twenty-four-hour food intake (monitored throughout the experiment) is shown at 1, 11, and 18 days post-injection (*dpi*) for control (*white bar*), 40 mg/kg STZ (low dose, *gray bar*), or 60 mg/kg STZ mice (high dose, *black bar*). Only intake at 1 dpi was significantly decreased. *Bottom panel*, Mouse body mass was examined throughout the experiment for control (*white circles*), 40 mg/kg STZ (*black square*), or 60 mg/kg STZ mice (*black triangle*). All values represent averages ± SE. *n* > 3 per condition; *p < 0.05 compared to control.

To test the effect of STZ dose on bone pathology, we injected 10-week-old male BALB/c mice with either 40 or 60 mg of STZ per kilogram body weight for 5 days. Control mice were injected with vehicle (citrate buffer) to control for the influence of any injection stress or buffer-induced effects on the animals. Monitoring of food intake throughout the study indicated a decrease only the day after the first STZ injection by 27% in the mice treated with 40 mg/kg and by 57% in the mice treated with 60 mg/kg streptozotocin (Figure 1). There were no significant changes at any other time point, although at later time points, food intake began to increase in the STZ-injected mice consistent with diabetic hyperphagia. Still, the both high- and low-dose STZ-treated mice lost weight, with the high dose group tending to lose more than the low-dose group (Figure 1).

**Figure 2:**
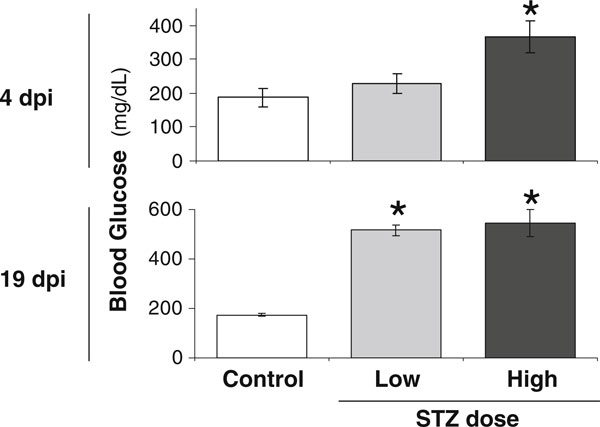
**High-dose STZ treatment causes a greater increase in blood glucose levels at early but not late time points compared to low dose STZ**. Shown are mouse blood glucose levels measured at 4 and 19 days post-injection (*dpi*) for control (*white bar*), 40 mg/kg STZ (low dose, *gray bar*), or 60 mg/kg STZ mice (high dose, *black bar*). Values represent the average ± SE. *n* > 3 per condition. *p < 0.05 compared to control.

In our standard protocol, elevated blood glucose levels (>300 mg/dl) observed at 12 days post-injection (dpi) are used to confirm diabetes in mice, while control mice exhibit blood glucose levels less than 200 mg/dl (not shown). In addition, we measured blood glucose levels at 4 and 19 dpi. Figure 2 demonstrates that at 19 dpi, both low and high STZ mice averaged blood glucose levels at or above 500 mg/dl, whereas control mice maintained normal non-fasting blood glucose levels less than 200 mg/dl. In contrast, at 4 dpi, only high-dose STZ mice exhibited significantly elevated blood glucose levels compared to controls. This suggests that the high-dose treatment induces a more rapid loss of insulin secretion (and therefore more rapid onset of diabetes) compared to low-dose treatment. Past studies indicate that the magnitude of STZ induction of serum glucose, urine volume, and glucosuria can, to some extent, correlate with dose concentration [25-27]. Along these lines, the effect of STZ on pancreatic beta cells has been shown to be dose-dependent by in vitro [28] and in vivo [26] studies. In the latter study, a greater number of pancreatic beta cells were shown to remain in mice after injection with multiple low doses of STZ (40 mg/kg for 5 days) compared to a single high-dose (200 mg/kg STZ) injection.

**Figure 3:**
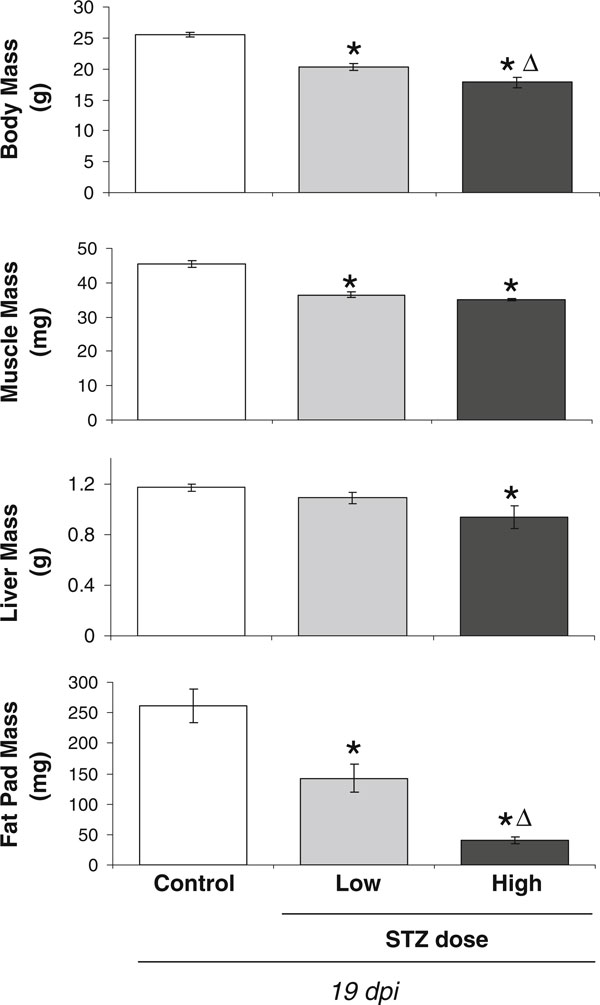
**Body, muscle and fat pad mass decreases with both low and high dose STZ treatment**. Body, muscle (tibialis anterior), liver, and fat pad (subcutaneous femoral fat) weights were measured at 19 dpi for control (*white bar*), 40 mg/kg STZ (low dose, *gray bar*), or 60 mg/kg STZ mice (high dose, *black bar*). Values represent the average ± SE. *n* > 3 per condition. *p < 0.05 compared to control; ^Δ^*p* < 0.05 compared to 40-mg/kg STZ dose.

Another indicator of the severity of diabetogenic and catabolic effects are changes in mouse body parameters (body, muscle, and fat pad mass), which were significantly decreased in both STZ-dosed groups compared to controls (Figure 3) as previously reported for STZ and spontaneously diabetic mice [17]. We also observed a decrease in liver weight that was only apparent in high-dose STZ mice and likely the result of reduced glycogen content. The high STZ dose also caused a significantly greater decrease in body and fat pad mass compared to the low STZ dose (Figure 3). Similarly, Van Dam et al. [29] and Howarth et al. [27] demonstrated a STZ dose-dependent decrease in rat body weight. The loss of fat pad mass that we observe is also consistent with reports demonstrating that high doses of STZ can directly cause adipocyte lipolysis [30]. The increased lipolysis can contribute to the modest but significant decrease in body weight compared to low-dose STZ-treated mice.

**Figure 4:**
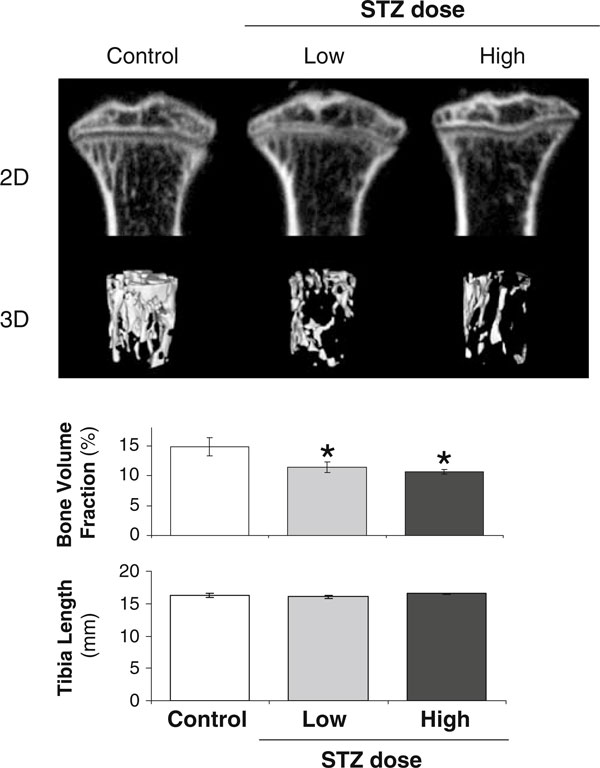
**Bone loss is evident in diabetes induced by both low- and high-dose STZ**. The two-dimensional images at the *top* were obtained from micro-computed tomography analyses of representative tibias from control and STZ-treated mice (40 days post first injection). Three dimensional images were composed of 760 consecutive slices. The *bar graphs* indicate the measured bone volume fraction (average ± SE) of the tibial trabecular region and the tibia lengths of control (*white bar*), 40 mg/kg STZ (low dose, *gray bar*), or 60 mg/kg STZ mice (high dose, *black bar*). *n* > 3 per condition. *p < 0.05 compared to control.

Next, we examined STZ dose effects on the skeleton. Bone density analyses at 19 dpi demonstrated, as expected, that STZ-treated mice lose tibial trabecular bone volume fraction (BVF) compared to control mice (Figure 4, images and graph). This is consistent with previous reports in pharmacologically induced and spontaneously diabetic mice and rats and is consistent with T1 diabetic human studies [2-4,12,16,17,31-50]. Interestingly, bone loss (in contrast to body and fat mass, Figure 3) was not dependent upon STZ dose as demonstrated by similar BVF between the low and high STZ groups. Our previous studies, in mice at 16–20 weeks of age, also demonstrate significant bone loss. Because the mice in the current study were 10 weeks old and can still exhibit skeletal growth (albeit at a slower rate than younger mice), we also examined bone length. No changes in tibial length were observed (Figure 4), suggesting that T1 diabetes did not alter bone growth in these mice.

**Figure 5:**
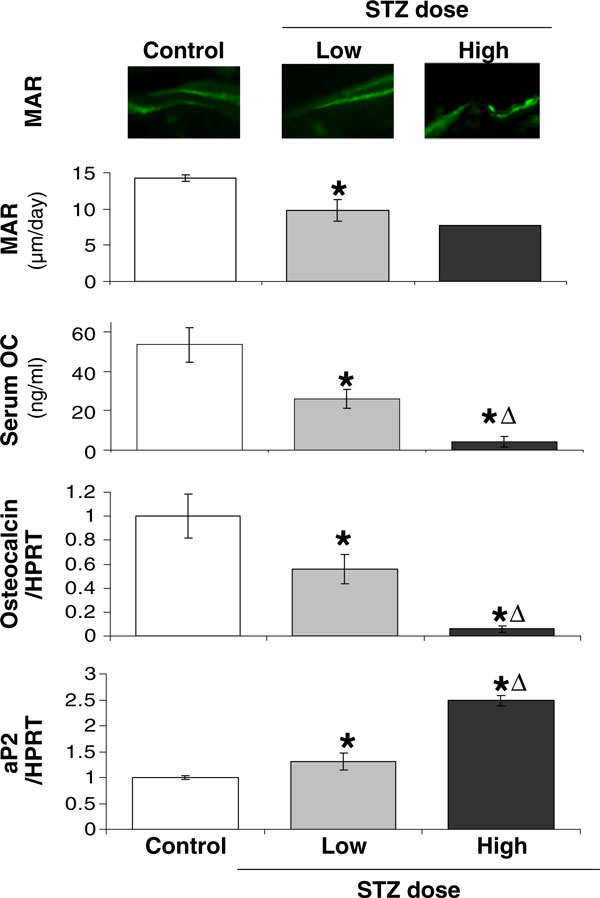
**STZ-induced T1-diabetes decreases MAR, serum osteocalcin, and bone osteocalcin mRNA while increasing aP2 expression**. Control (*white bar*), 40 mg/kg STZ (low dose, *gray bar*), and 60 mg/kg STZ mice (high dose, *black bar*) were examined at 19 dpi for osteoblast parameters: mineral apposition rate (*MAR*; representative fluorescent images of calcein labeling of bone shown at *top of figure*), serum osteocalcin (*OC*), and tibial osteocalcin mRNA levels [expressed relative to hypoxanthine–guanine phosphoribosyl transferase (*HPRT*), a housekeeping gene). Expression of a mature adipocyte marker, aP2, was also examined. For all RNA analyses, expression was expressed relative to control levels which were set to 1. Values represent the average ± SE. *n* > 3 per condition, except for high STZ dose MAR (*n* = 1). *p < 0.05 compared to control; ^Δ^*p* < 0.05 compared to 40-mg/kg STZ dose.

Previously, we demonstrated that low-dose STZ suppresses osteoblast markers and activity, so we examined the influence of STZ dose/diabetes severity on this effect. First, we measured mineralization apposition rate. Figure 5 demonstrates that this functional measure of osteoblast activity (bone formation) is decreased in both low and high STZ dose diabetic mice. Consistent with this finding, serum osteocalcin levels were decreased in STZ mice, but to a much greater extent in high- versus low-dose mice. To further assess effects on osteoblasts, RNA analyses were done to examine gene expression, which we have found to be an even more sensitive marker for determining early responses in bone. As shown in Figure 5, osteocalcin (OC) messenger RNA (mRNA) levels, a marker of mature osteoblasts, are significantly decreased in whole tibias of T1 diabetic mice compared to controls. High-dose STZ caused a further tenfold reduction in OC RNA levels compared to low-dose STZ-treated mice, suggesting a relationship to disease severity. The suppression of osteocalcin expression at the mRNA and/or protein (serum) level has been seen previously in STZ and spontaneously diabetic mouse and diabetic rat models [17,33,36,39,46,49]. However, to our knowledge, this has not been shown to be STZ dose/disease severity dependent. Previously, we demonstrated a positive correlation between changes in OC mRNA levels and osteoblast activity (bone density) [16,17,37,51]. However, despite a large difference in OC mRNA levels (Figure 5), we did not observe a significant difference in BVF between the two doses of STZ (Figure 4). We suspect that the differences in OC mRNA between low and high STZ dose will continue to impact BVF at later time points since there would be a delay between changes in osteoblast activity and actual BVF changes. These RNA findings suggest the utility of gene expression studies in bone as early indicators of later changes in function.

T1 diabetic bone loss is associated with increased marrow adiposity. Therefore, we examined the mRNA levels of a marker for mature adipocytes, adipocyte binding protein 2 (aP2, also known as fatty acid binding protein-4), which correlates with the extent of bone marrow adiposity [16,17,37,51]. Similar to previous reports in STZ-induced diabetic mice and spontaneously diabetic mice [16,17,37,51], either dose of STZ increased aP2 expression in bone, but the magnitude of this increase was significantly greater in mice treated with the higher dose of STZ (Figure 5).

Table 1 demonstrates the similarity of STZ effects on bone to those of a spontaneous T1-diabetic mouse model, NOD mice. All diabetic mice (spontaneous, STZ low dose, STZ high dose) exhibit significant bone loss, decreased osteocalcin expression, and increased aP2 compared to experimental non-diabetic control mice. However, there were differences in the magnitude of changes. In this study, we examined STZ-treated mice at 19 dpi, whereas in our past study in NOD mice, we examined mice at 40 dpi [17]. The latter study included the multiple low-STZ-dose model which demonstrated a 50% suppression in BVF at 40 dpi corresponding with changes in NOD mice. Thus, bone loss at 40 dpi is greater than at 19 dpi. With regard to RNA, osteocalcin exhibited a 55% decrease in low-STZ-dose mice, a 70% decrease in diabetic NOD mice, and a 94% decrease in high-STZ-dose mice compared to controls. The latter large suppression in osteocalcin expression has not been previously observed. aP2 expression was increased in all models, but high-STZ-dose mice exhibited less than half the levels seen in NOD mice, and low STZ dose exhibited even less elevation than high dose. We have previously observed some variability in the magnitude of aP2 induction in the low-STZ mice, suggesting that multiple factors, including hyperlipidemia, inflammation, age (we examined 13 weeks mice compared to greater than 24 weeks NOD mice), that can differ between experiments may contribute to the magnitude of adiposity in diabetic bone. Still, despite all these differences, both spontaneous and STZ-induced mouse models lose bone, have decreased osteoblast function and marrow adiposity.

**Table 1:** Bone phenotype of spontaneous versus pharmacologic T1-diabetic mouse models

	40 dpi	19 dpi—STZ dose	
	
Percentage of control	NOD (%)	Low (%)	High (%)
BVF	50	76	69
OC RNA	30	45	6
aP2 RNA	570	140	248

Average diabetic mouse values are expressed as a percent of non-diabetic mouse values. Differences were all statistically significant (compared to controls)

**Figure 6:**
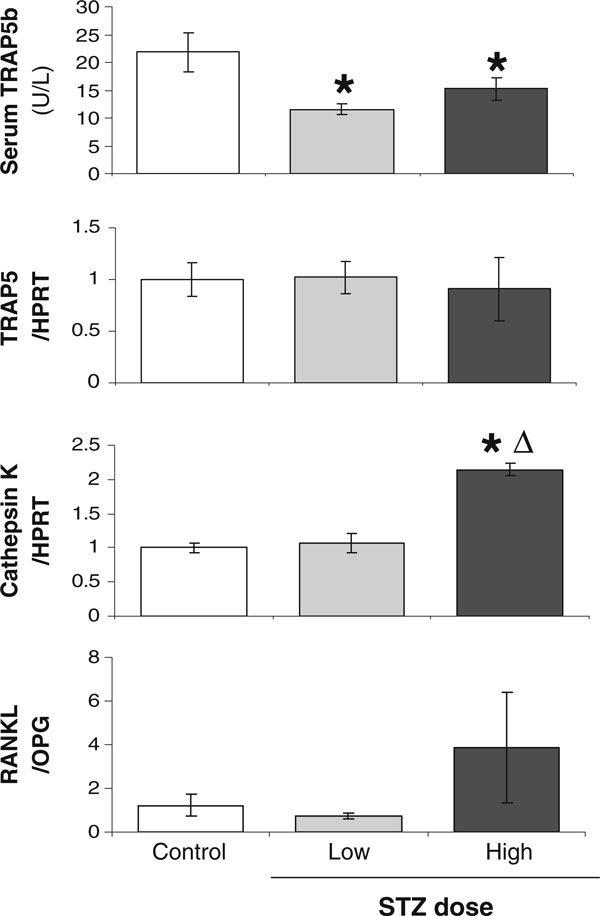
**T1-diabetes decreases serum TRAP5b, while high-dose STZ increases cathepsin K and RANKL/OPG ratio**. Control (*white bar*), 40 mg/kg STZ (low dose, *gray bar*), and 60 mg/kg STZ mice (high dose, *black bar*) were examined at 19 dpi for osteoclast markers and activity: serum TRAP5b, TRAP5 RNA, cathepsin K RNA, and RANKL/OPG RNA ratio. RNA levels were expressed relative to HPRT. RNA values represent the average ± SE and are expressed relative to control mice (set to 1). *p < 0.05 compared to control. ^Δ^*p* < 0.05 compared to 40-mg/kg STZ dose.

Next we examined dose/severity effects on markers of osteoclast maturation and activity. Levels of serum active TRAP5b, a marker of active osteoclasts, were decreased in both STZ doses (Figure 6), consistent with previous studies suggesting that resorption is not increased in T1 diabetic humans and rodent models [2,16,33,40,43,45,51-63]. We measured serum osteoclast markers rather than urine markers because diabetes is associated with polyuria (which can confound measurements due to large differences in urine volume between control and diabetic mice) and can potentially result in nephropathy which could affect urine levels of measured factors. We also examined the expression levels of osteoclast markers in whole bone RNA. Figure 6 demonstrates that TRAP5 mRNA levels did not differ between conditions, although it showed a trend to decrease in high-STZ-treated mice. Differences between RNA and serum levels may result from differences between RNA and protein stability and between local and systemic measures (the later would be the sum of changes in all bones which could result in significant differences). Interestingly, we found that cathepsin K, a protease found in lysosomes and active in osteoclast bone resorption, was significantly increased in high-dose STZ mice only. Similarly, the RANKL to OPG ratio is also increased in high-STZ mice, supporting that under high dose/severity, a local environment may exist that could promote resorption. The fact that serum TRAP5b levels are not elevated suggests that under these conditions, osteoclasts are not able to be fully activated. This may stem from additional factors in T1 diabetes, such as high glucose and advanced glycosylation end products, suppressing osteoclast activation [64,65]. There are some studies that support an increase in osteoclast activity in diabetic rodent models [66,67]. Our data suggest that differences in resorptive markers measured and in disease severity could contribute to differences seen between studies (in addition to species, gender, and age differences).

The exact mechanisms accounting for differences in the magnitude of low- versus high-STZ dose effects are not fully known, but our data indicate that there are differences in disease onset (based on earlier blood glucose elevation in high dose mice) and metabolic/catabolic effects (a greater loss of body and fat pad mass in high-dose mice) between the two dosing regimes. The greater loss of fat pad mass in high-dose STZ mice is not only a disease severity issue. Fat pad loss could cause a proportional decrease in serum adipokine levels such as leptin, as previously demonstrated in low-STZ-dose mice [37,68]. In addition, greater loss of fat pad mass could lead to a greater increase in serum fatty acids which could in turn stimulate PPARγ2 and induce adipocyte maturation in the bone marrow at the expense of osteoblast maturation. This possibility is supported by a study where 55 or 100 mg/kg STZ or alloxan was administered to rats and it was found that only the high dose of either drug caused increased serum triglycerides and cholesterol [69].

**Figure 7:**
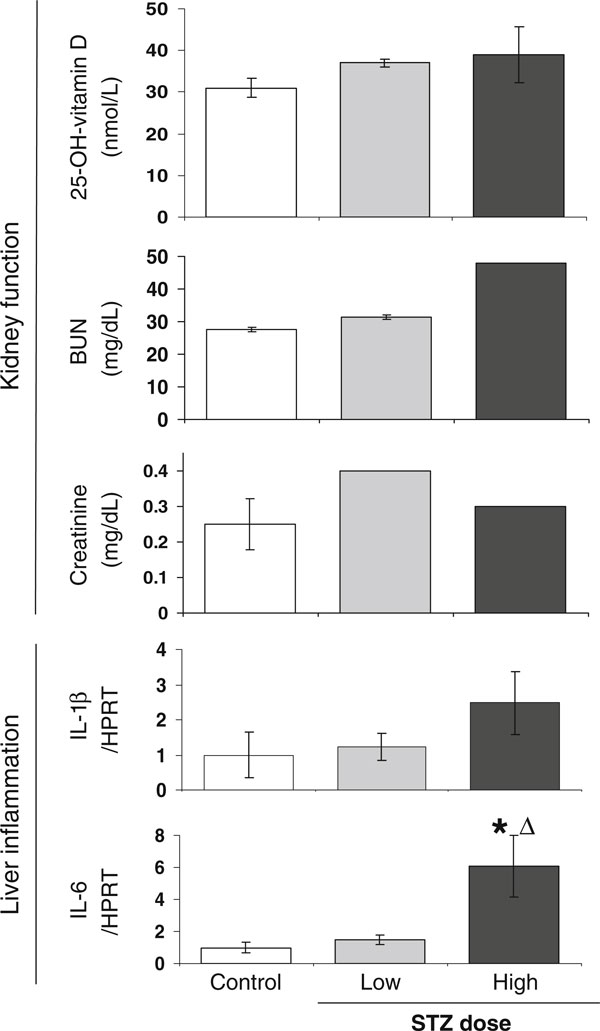
**Both low and high STZ dose does not influence kidney function, but high dose is associated with liver inflammation**. Control (*white bar*), 40 mg/kg STZ (low dose, *gray bar*), and 60 mg/kg STZ mice (high dose, *black bar*) were examined at 19 dpi for markers of kidney function (25-OH vitamin D, BUN, and creatinine serum levels) and liver toxicity (IL-1β and IL-6 RNA levels). For serum analyses, samples were pooled to meet the quantities necessary for measurement. For 25-hydroxyvitamin D: three samples were analyzed per condition (each sample contained serum pooled from one to four individual mice). BUN and creatinine analyses were done on one (high dose) or two (control and low dose) blood samples, each containing serum pooled from five individual mice. IL-6 and C/EBPβ RNA levels were expressed relative to HPRT. Serum values represent mean ± SD. RNA values represent the average ± SE and are expressed relative to control mice (set to 1). *p < 0.05 compared to control; ^Δ^*p* < 0.05 compared to 40-mg/kg STZ dose.

It is possible that high doses of STZ are in general more toxic to the mice. In monkeys, a high dose (100 mg/kg) of STZ was found to elevate kidney and liver function tests, whereas low-dose (55 mg/kg) STZ-treated animals had normal kidney and liver function tests [70]. Both doses effectively induced diabetes, but the low dose did not appear to exhibit toxic effects. Chronic kidney disease (CKD) is a common complication of diabetes and can also lead to decreased osteoblast and osteoclast function [71]. Patients and animal models with CKD and nephrotoxicity have increased BUN, creatinine, and low vitamin D [71,72]. Therefore, we measured 25-hydroxy-vitamin D, BUN, and creatinine levels in the control, low-, and high-STZ-dosed mice. There were no differences between groups, suggesting that at the STZ doses we used, kidney damage is not apparent and therefore does not contribute to the bone phenotype we observed in low- or high-dose STZ models (Figure 7). We also examined liver cytokine expression, which can be a sign of liver toxicity [73], and found that only high STZ dose treatment caused a significant increase in IL-6 and a trend to increase for IL-1β (Figure 7). The potential of liver toxicity is consistent with reduced liver mass (Figure 3) seen only in high-STZ-dosed mice.

Taken together, this report demonstrates that multiple low-dose STZ-induced T1 diabetic mice exhibit (1) a decrease in body, muscle, and fat pad mass, (2) decreased bone volume, (3) decreased osteoblast markers and function, (4) increased marrow adiposity, (5) a similar bone phenotype to spontaneous mouse models, (6) decreased serum active TRAP5b, and (7) no change in kidney and liver function markers. More importantly, bone loss and decreased osteocalcin protein are also observed in T1 diabetic patients [2-4,12,16,17,31-50]. High-dose STZ mice exhibit more catabolic effects on body parameters, suggesting increased disease severity (and secondary effects such as liver inflammation). This may play a role in the elevation of some osteoclast parameters (which are not increased in the low dose model). While both doses cause bone loss, it is important to correctly choose the dosing regime to be used to obtain consistent results. Dose response differences may contribute to differences in the literature and can be capitalized upon and used as a tool to provide insight into mechanisms regulating changes in bone that are dependent upon disease severity.

## Appendix

### Protocols

#### Streptozotocin Injections

1. Make 0.1 M citrate buffer pH 4.5. For 100 ml, combine 1.05 g citric acid and 1.48 g sodium citrate in dH_2_0. Ensure pH is 4.5, then bring up to 100 mL with water.

2. Determine weight of the mice and calculate mass of streptozotocin (Sigma S0130) needed for injections of 100 ml per mouse.

3. Quickly weigh streptozotocin, dissolve in cold citrate buffer, and filter sterilize through a 22-μm pore filter.

4. Immediately draw into a 1.0-mL syringe and inject intraperitoneally into mouse. Speed is imperative because streptozotocin degrades rapidly once dissolved.

5. Repeat everyday for 5 days total.

6. Maintain a mouse health score sheet (monitoring body weight, polyurea, signs of stress, eating habits) regularly throughout the course of the study.

7. Seven days after the final injection, check fed blood glucose of the animal.

#### Determination of Blood Glucose

1. Hold the mouse firmly by the skin of the back/neck.

2. Shave the inner thigh of one of the hind legs.

3. Put a small amount of petroleum jelly on the shaved area to help visualize the saphenous vein.

4. Prick the saphenous vein with a 25-gauge needle. A small drop of blood should form.

5. Measure the blood glucose with an AccuChek Compact glucometer (Roche).

6. If the blood glucose is greater than 300 mg/dL, the mice are diabetic. This day is then often considered day 0.

7. Hold clean gauze over the site of the blood draw until bleeding has stopped.

#### Tissue Isolation

1. Wait until the desired duration of diabetes has passed and euthanize the mice by an approved method.

2. Measure blood glucose immediately after euthanasia if desired or collect serum for a glucose assay.

3. Remove desired organs and bones.

4. Quickly clean bones for RNA or protein extraction of all surrounding muscle and connective tissue and freeze in liquid nitrogen.

5. Fix bones for micro-computed tomography analyses in formalin. Transfer to 70% EtOH after 24 h.

#### RNA Extraction from Tibia

1. Crush tibia in a Bessman tissue pulverizer under liquid nitrogen conditions.

2. Put crushed bone into a 2.0 mL flat bottom microcentrifuge tube.

3. Add 1.0 mL TRI reagent solution (Molecular Research Center, Inc.).

4. Immediately homogenize and place on ice (Omni International TH homogenizer, Marietta, GA, USA).

5. Continue with RNA extraction according to TRI reagent protocol.

6. Make cDNA with 2.0 mg RNA, SuperScript II reverse transcriptase kit, and oligo dT_(12–18)_ primers (Invitrogen) according to the manufacturer's protocol.

7. Amplify 1.0 μL cDNA to a final volume of 25 mL by PCR with iQ SYBR Green Supermix (Bio-Rad) and 10 pmol of desired primer.
